# Multiclass Support Vector Machine-Based Lesion Mapping Predicts Functional Outcome in Ischemic Stroke Patients

**DOI:** 10.1371/journal.pone.0129569

**Published:** 2015-06-22

**Authors:** Nils Daniel Forkert, Tobias Verleger, Bastian Cheng, Götz Thomalla, Claus C. Hilgetag, Jens Fiehler

**Affiliations:** 1 Department of Radiology and Hotchkiss Brain Institute, University of Calgary, 3330 Hospital Drive NW, Calgary, AB T2N 4N1, Canada; 2 Department of Diagnostic and Interventional Neuroradiology, University Medical Center Hamburg-Eppendorf, Martinistr. 52, 20246, Hamburg, Germany; 3 Department of Neurology, University Medical Center Hamburg-Eppendorf, Martinistr. 52, 20246, Hamburg, Germany; 4 Department of Computational Neuroscience, University Medical Center Hamburg-Eppendorf, Martinistr. 52, 20246, Hamburg, Germany; 5 Department of Health Sciences, Boston University, 635 Commonwealth Ave, Boston, MA, 02215, United States of America; INSERM U894, FRANCE

## Abstract

**Purpose:**

The aim of this study was to investigate if ischemic stroke final infarction volume and location can be used to predict the associated functional outcome using a multi-class support vector machine (SVM).

**Material and Methods:**

Sixty-eight follow-up MR FLAIR datasets of ischemic stroke patients with known modified Rankin Scale (mRS) functional outcome after 30 days were used. The infarct regions were segmented and used to calculate the percentage of lesioned voxels in the predefined MNI, Harvard-Oxford cortical and subcortical atlas regions as well as using four problem-specific VOIs, which were identified from the database using voxel-based lesion symptom mapping. An overall of 12 SVM classification models for predicting the corresponding mRS score were generated using the lesion overlap values from the different brain region definitions, stroke laterality information, and the optional parameters infarct volume, admission NIHSS, and patient age.

**Results:**

Leave-one-out cross validations revealed that including information about the stroke location in terms of lesion overlap measurements led to improved mRS prediction results compared to classification models not utilizing the stroke location information. Furthermore, integration of the optional features led to improved mRS prediction results in all cases tested. The problem-specific brain regions and additional integration of the optional features led to the best mRS predictions with a precise multi-value mRS prediction accuracy of 56%, sliding window multi-value mRS prediction accuracy (mRS±1) of 82%, and binary mRS (0-2 vs. 3-5) prediction accuracy of 85%.

**Conclusion:**

Therefore, a graded SVM-based functional stroke outcome prediction using the problem-specific brain regions for lesion overlap quantification leads to promising results but needs to be further validated using an independent database to rule out a potential methodical bias and overfitting effects. The prediction of the graded mRS functional outcome could be a valuable tool if combined with voxel-wise tissue outcome predictions based on multi-parametric datasets acquired at the acute phase.

## Introduction

Acute ischemic stroke diagnosis and treatment decision is mostly based on clinical parameters such as severity of the clinical deficit at admission, e.g. assessed by the National Institutes of Health Stroke Scale (NIHSS), patient age, comorbidities, and image-based features derived from multi-parametric CT or MR imaging. Within this context, diffusion- (DWI) and perfusion-weighted (PWI) MRI have been in the focus of research for several years [1], since combination of these two image sequences allows identifying the PWI-DWI mismatch, which is a surrogate of the ischemic penumbra region in stroke patients and represents the tissue-at-risk and target for any therapy. This goal can, for example, be achieved by simple thresholding of the DWI dataset and a single perfusion parameter map, mostly the Tmax or time-to-peak parameter map, derived from the PWI dataset [2–4] or by using more sophisticated multi-parametric tissue outcome predictions on a voxel-level using statistical methods or machine learning techniques [5–8].

Regardless of the method used for predicting the final tissue outcome, the prediction result represents only an approximation of the future lesion size and location to be expected without any direct information about the associated functional outcome. Within this context, a small lesion can be associated with a worse functional outcome compared to larger lesions, which are located in a brain region that is less important regarding the functional outcome score or that may exhibit a higher potential to compensate lesions. The question if and how the lesion size and location relate to functional outcome is still not well understood.

Several methods for predicting the functional outcome after an ischemic stroke using clinical and image-based features have been proposed in the past. However, most previously described methods have in common that only a dichotomized outcome (good vs. severe) is predicted and logistic regression modelling instead of potentially more powerful high-level machine learning techniques is used. Furthermore, information about the lesion location within the brain has rarely been used for functional outcome prediction although it has been shown that it is an important determinant of functional stroke outcome [9].

The aim of this study was to evaluate the feasibility of a graded functional outcome prediction after ischemic stroke using a multi-class support vector machine (SVM) and information about the size and spatial distribution of the final stroke lesion. Within this context, the precision of the predicted functional outcome is compared for twelve SVM models incorporating different image-based and clinical parameters to identify the optimal feature set for the prediction of the functional outcome.

## Material and Methods

### 2.1. Patients and MR protocol

The local study database was retrospectively screened for datasets of patients with an acute ischemic stroke according to the following inclusion criteria:

1. A present infarction in the territory of the middle cerebral artery (MCA), and no previous strokes or previous functional disability (mRS = 0).
2. Available clinical information about patient age, and NIHSS at admission.
3. Available modified Rankin Scale (mRS) functional outcome data at approximately 30 days after stroke symptom onset determined by an experienced neurologist using a structured interview of the patient or next of kin.
4. Available follow-up Fluid Attenuated Inversion Recovery (FLAIR) MR image sequence corresponding to mRS functional outcome assessment.

The modified Rankin Scale (mRS) [10,11] is a measure of global disability devised for clinician-reported evaluation of stroke patient outcomes and is commonly used for follow-up functional outcome assessment in clinical trials. The mRS grades the global disability in the daily life of stroke patients, with a predominance of motor functions. It is defined between 0 and 6 points, where a mRS of 0 means that no symptoms for disability are present, 5 describes most severe disabilities, and 6 denotes that the patient did not survive. Based on the identified patient cases meeting all criteria, 68 datasets were randomly selected so that the mRS scores were as equally distributed among the possible mRS range between 0 and 5 as possible. This approach was chosen since a balanced group size distribution reduces the problem of a potential bias in the learning phase of the subsequent classification process. This goal was achieved for all mRS scores from 0 to 4 with 12 patient cases identified for each score. However, only 8 patients with mRS 5 could be identified, which can be ascribed to the fact that this score is diagnosed seldom and follow-up imaging is often not available or does not display a proper quality due to motion artefacts. Also, patients with mRS 6 were not be included in this study as this score describes that the patient did not survive the stroke and, thus, no image information is available for these cases. The study was approved by the local ethics committee and institutional review board (University Centre Hamburg-Eppendorf, Germany). Written informed consent was obtained from all patients. All examinations were conducted according to the Declaration of Helsinki.

All magnetic resonance imaging (MRI) measurements were performed on a 1.5T Sonata or Avanto MRI scanner (both Siemens, Erlangen, Germany). An FLAIR MR image sequence was used for definition of the final infarct lesion in each patient. The typical parameters for acquisition of the FLAIR image sequence were as follows: TE = 108 ms, TR = 7900 ms, TI = 2500 ms, flip angle = 150°, and a spatial resolution of 0.45 x 0.45 x 5 mm³.

### 2.2. Image processing

In order to relate the information about the stroke volume and location to the clinical mRS information, the corresponding lesions have to be segmented in each follow-up FLAIR dataset, which was performed using the software tool AnToNIa [12]. In contrast to the lesion volume, which can be determined directly from this lesion segmentation, the quantitative analysis to which extent a certain brain region is affected by a given lesion requires a previous definition of the brain regions of interest within the patients. Therefore, the MNI brain atlas [13], for which several structural or functional brain region definitions are available, was used for all analysis procedures described in the following.

Briefly, the MNI brain atlas was registered to each patient dataset by calculating the optimal affine transformation to the patient dataset by maximization of the mutual information [14], and applying a linear interpolation. After registration of the MNI brain atlas, the corresponding brain regions, as defined in the MNI brain atlas, were transformed to the patient dataset by applying a nearest neighbor interpolation and the overlap between the defined final infarct lesion and each brain region was quantified.

### 2.3. Volume-of-Interest definition

Four different brain region definitions were used in this work and compared against each other regarding the potential of predicting the associated graded mRS score. First, the nine standard MNI structural brain regions defined in the MNI atlas were used to determine the regional percentage of lesioned voxels in each brain region within a stroke patient. Likewise, the Harvard-Oxford cortical and subcortical structural atlases were used in the same fashion for lesion overlap quantification. Details about the brain regions included in the two Harvard-Oxford atlases are provided in the online appendix (S1 Table).

These predefined atlas brain regions might not reflect the optimal choice for predicting a functional outcome corresponding to a volumetric infarct lesion because uninformative or redundant features may downgrade the classification performance. To investigate and overcome this potential challenge, four problem-specific atlas brain regions were identified as an alternative to the simple predefined atlas brain regions by employing a method derived from voxel-based lesion symptom mapping (VLSM) [15–17].

**Fig 1:**
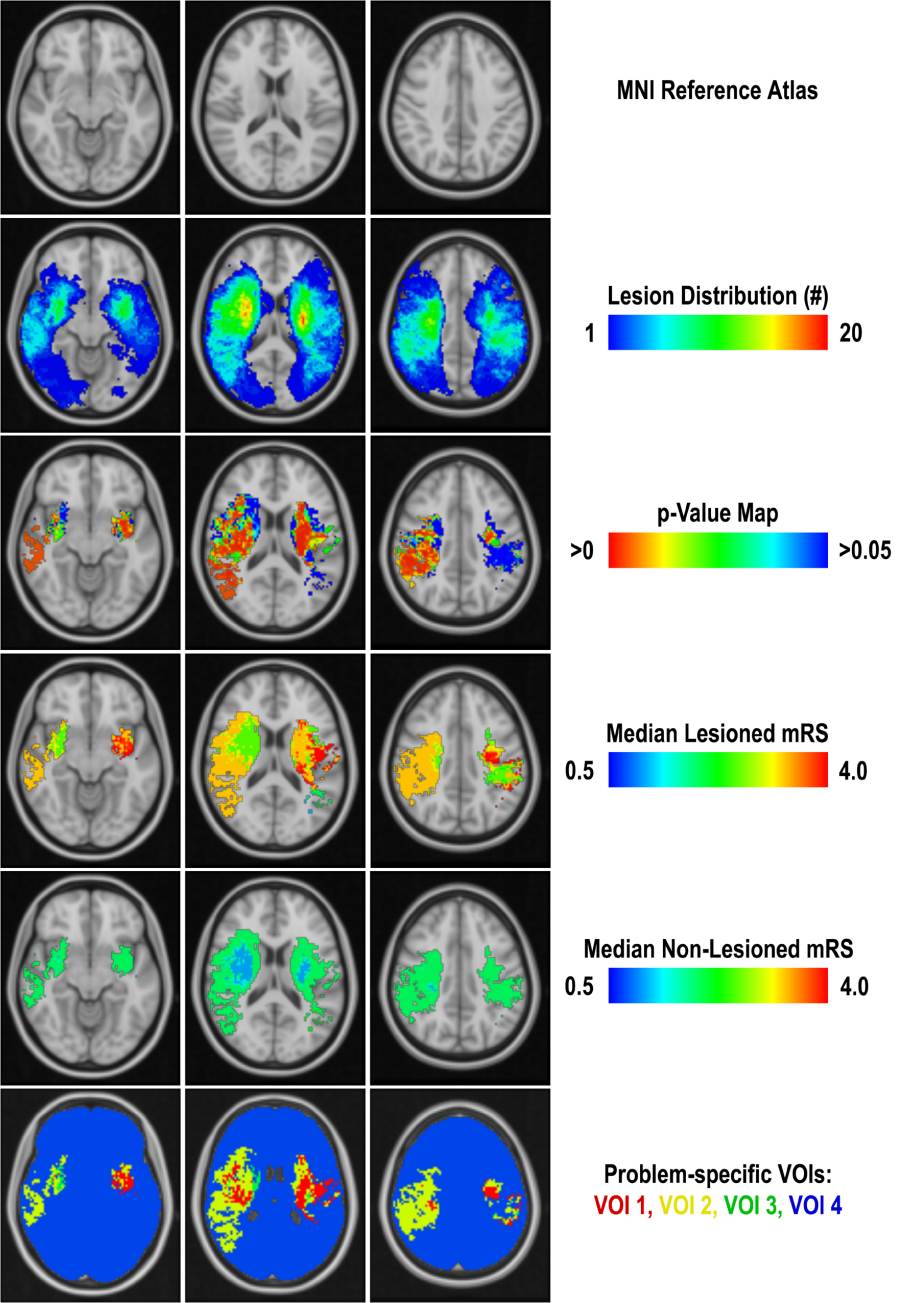
Illustration of the single processing steps used for generation of the problem-specific brain regions in three selected slices. From top to bottom: MNI reference atlas, infarct distribution map used to exclude voxels lesioned in less than five patients from statistical calculations, p-value map used to exclude voxel with a significance level p≥0.05 from the VOI generation, median mRS values of lesioned and non-lesioned voxels used to define the final VOIs based on the median mRS difference d (VOI1: d > 2, VOI2: 1 < d ≤ 2, VOI3: 0 < d ≤ 1, VOI4: remaining voxels).

For this purpose, the affine transformations determined by registering the atlas to each patient dataset were inverted and used to transform the individually segmented lesions into the MNI atlas space employing a nearest-neighbour interpolation. After transformation of all lesion segmentations into atlas space, patients were separated for each voxel within the brain tissue into a lesion group and non-lesion group, respectively. After group separation for each voxel, the corresponding mRS scores of the patients were used to calculate the voxel-wise significance level (p-value) as well as the t-score employing a two-sided t-test. Furthermore, the median mRS score was determined for both groups and used to calculate the corresponding median mRS score difference d between the two groups for each voxel (Fig 1). These calculations were performed separately for patients with left- and right-hemispheric infarcts. Since the number of patients in the intact and lesion groups varies for each voxel, a minimum group size of 5 patients was arbitrarily defined to be required for performing the voxel-wise statistics.

After calculation of the statistical maps, four problem-specific brain regions were determined based on the voxel-wise statistical information. For this purpose, only voxels with a significance level p<0.05 were included for generation of the volumes-of-interest (VOIs). All voxels exhibiting a median mRS difference d > 2 were included in the first VOI, voxels exhibiting a median mRS difference 1 < d ≤ 2 were included in the second VOI, and all voxels exhibiting a 0 < d ≤ 1 were included in the third VOI. Finally, all remaining voxels within the brain tissue including those with a significance level p ≥ 0.05 and those excluded due to the minimum group size criterion were combined to the fourth VOI (Fig 1).

The main idea of this procedure was to model that certain brain regions are more important for the functional outcome than others and, thus, to include this information in the mRS prediction model. It was hypothesized that already a small fraction of lesioned voxels in the first VOI lead to a severe functional outcome while the fraction of lesioned voxels needs to be higher for the second VOI, even higher for the third VOI, and highest for the fourth VOI to result in the same functional outcome severity.

### 2.4. Classification and evaluation

The quantitative lesion overlap values for each VOI of the predefined brain regions (MNI, Harvard-Oxford cortical, and Harvard-Orxford subcortical), as well as for the four problem-specific brain VOIs were separately used for training of classification models for the lesion-based prediction of the corresponding mRS scores. Furthermore, the t-score map resulting from VLSM was also transformed to each patient dataset employing a linear interpolation and used to calculate the sum of the voxel-wise t-scores within the lesion, which was then used for training of a separate classification model.

The multi-class SVM described by Crammer and Singer [18], which is implemented in the LIBLINEAR toolbox [19], was used in this work. The cost parameter C, which controls the trade-off between training errors and SVM model complexity, was empirically optimized for each of the twelve classification models by maximizing the area under the receiver operating characteristic curve (ROC AUC).

A total of twelve SVM models, which are summarized in Table 1, were generated by supervised training. The simple model and the extended simple model were generated so that the potential benefit of including information about the stroke location in addition to the lesion volume can be evaluated. By contrast, the eight models using the overlap measurements enable to analyse if the predefined or problem-specifically defined VOIs are better suited for the lesion-based mRS prediction and if the additional integration of the optional parameters improves the prediction accuracy. Finally, the two VLSM models enable to investigate the potential of a direct application of the VLSM map for the lesion-based mRS prediction instead of using it as the basis for definition of the problem-specific brain regions. The lesion side feature was included in all SVM models using lesion overlap measurements to account for different volumes of corresponding brain region VOIs in the left and right hemisphere, which is especially important for the problem-specific VOIs.

**Table 1:** Overview of the features used for generation of the twelve SVM models. The complete Harvard-Oxford cortical brain structure list can be found in S1 Table.

**Simple Model:** Lesion Volume	**Extended Simple Model:** Lesion Volume, Lesion Side, Patient Age, Admission NIHSS
**MNI Model:** % Lesioned Caudate, % Lesioned Cerebellum, % Lesioned Insula, % Lesioned Putamen, % Lesioned Thalamus, % Lesioned Frontal Lobe, % Lesioned Occipetal Lobe, % Lesioned Parietal Lobe, % Lesioned Temporal Lobe, Lesion Side	**Extended MNI Model:** % Lesioned Caudate, % Lesioned Cerebellum, % Lesioned Insula, % Lesioned Putamen, % Lesioned Thalamus, % Lesioned Frontal Lobe, % Lesioned Occipetal Lobe, % Lesioned Parietal Lobe, % Lesioned Temporal Lobe, Lesion Side, Lesion Volume, Patient Age, Admission NIHSS
**Harvard-Oxford Subcortical Model**: % Lesioned Cerebral White Matter, % Lesioned Cerebral Cortex, % Lesioned Thalamus, % Lesioned Caudate, % Lesioned Putamen, % Lesioned Pallidum, % Lesioned Hipposcampus, % Lesioned Amygdala, % Lesioned Accumbens, % Lesioned Brain Stem, Lesion Side	**Extended Harvard-Oxford Subcortical Model:** % Lesioned Cerebral White Matter, % Lesioned Cerebral Cortex, % Lesioned Thalamus, % Lesioned Caudate, % Lesioned Putamen, % Lesioned Pallidum, % Lesioned Hipposcampus, % Lesioned Amygdala, % Lesioned Accumbens, % Lesioned Brain Stem, Lesion Side, Lesion Volume, Patient Age, Admission NIHSS
**Harvard-Oxford Cortical Model:** % Lesioned Frontal Pole, % Lesioned Insular Cortex, …, Lesion Side	**Extended Harvard-Oxford Cortical Model:** % Lesioned Frontal Pole, % Lesioned Insular Cortex, …, Lesion Side, Lesion Volume, Patient Age, Admission NIHSS
**Problem-specific Model:** % Lesioned VOI1, % Lesioned VOI2, % Lesioned VOI3, % Lesioned VOI4, Lesion Side	**Extended Problem-specific Model:** % Lesioned VOI1, % Lesioned VOI2, % Lesioned VOI3, % Lesioned VOI4, Lesion Side, Lesion Volume, Patient Age, Admission NIHSS
**VLSM Model:** Lesion t-score sum, Lesion Side	**Extended VLSM Model:** Lesion t-score sum, Lesion Side, Lesion Volume, Patient Age, Admission NIHSS

A leave-one-out cross validation was performed to evaluate the accuracy of each classification model. To prevent a potential bias due to inclusion of a dataset to be predicted in the calculation of the problem-specific brain regions, the leave-one-out cross evaluation included also the definition of these atlas regions. More precisely, the problem-specific atlas regions were generated individually for each patient employing the procedure described above using the 67 other datasets. Thus, the problem-specific brain regions as well as the t-score maps used as the basis for mRS prediction differed slightly for each patient since the patient to be classified was excluded from the t-score map calculation and definition of the problem-specific brain regions.

The accuracy of the lesion-based mRS prediction using the twelve SVM classification models was quantified by three accuracy metrics. First, the ***exact accuracy*** was calculated by comparing the predicted mRS score with the ground truth mRS score (ranging from 0 to 5). Only exact mRS predictions compared to the ground truth classification were counted as correct for this accuracy measure. In the second evaluation metric, the ***sliding-window accuracy***, a mRS score ±1 compared to the ground truth classification was counted as correct. For example, a predicted mRS score of 2 compared to a ground truth mRS score of 3 would count as correct for the sliding-window accuracy metric but not for the exact accuracy measure. Finally, the predicted as well as ground truth mRS scores were binarized into a favourable outcome group (mRS 0–2) and a severe outcome group (mRS 3–5), which were then used to calculate the ***binary accuracy*** measure by comparing the predicted binarized mRS outcome with the ground truth binarized mRS functional outcome. Furthermore, Bland-Altman plots [20] were generated for each classification model to analyse the agreement between the ground-truth and estimated mRS.

The generated SVM models lack of an intuitive explanatory value regarding the classification decisions made. This means that the resulting classifications are not as easily explainable compared to other classification techniques such as decision trees. Therefore, an additional statistical evaluation of the lesion-overlap measures as calculated for the predefined MNI and Harvard-Oxford cortical and subcortical brain regions as well as for the problem-specifically defined VOIs was performed, using Spearman’s rank correlation coefficient to identify those brain regions that correlate with the mRS score. Here, only brain regions that were affected by a lesion in a patient were considered for this calculation, while non-affected brain regions were excluded, to avoid a strong impact of non-lesioned brain regions on the correlation metric.

## Results

### 3.1. Patient characteristics

Table 2 shows the characteristics of the 68 patients included in this study. Overall, the different mRS patient groups were comparable regarding the lesion side, age, and days until follow-up imaging. In contrast to this finding, the lesion volume and the NIHSS at admission are both increasing with severity of the functional outcome measured by mRS.

**Table 2:** Patient characteristics for the different mRS patient groups.

Group	Gender (w/m)	Side (l/r)	Median Age in years	Median Admission NIHSS	Lesion Volume in mL (±std)	Median Follow-up imaging time in days
mRS 0	2/10	7/5	75	11.5	7.04±5.39	32
mRS 1	5/7	6/6	72.5	8	5.98±5.88	32.5
mRS 2	8/4	7/5	62	12.5	14.95±12.96	35.5
mRS 3	5/7	6/6	64	15.5	33.14±22.97	28
mRS 4	6/6	5/7	72	17.5	60.64±59.11	37.5
mRS 5	3/5	6/2	73.5	18	100.15±80.98	39.5
**All**	**29/39**	**37/31**	**71.5**	**13**	**33.27±48.67**	**34.5**

### 3.2. mRS prediction results

The overall results of the leave-one-out cross evaluation of the different classification models and corresponding cost parameters used for each model are given in Table 3. The complete confusion matrix for each model, in which each column represents the predicted mRS scores and each row the ground truth mRS scores, can be found in S1 Data.

**Table 3:** Quantitative results of the leave-one-out cross evaluation of the twelve SVM models.

Model	Exact accuracy	Sliding-window accuracy	Binary accuracy
**Simple Model (C = 2.75)**	23.53%	57.35%	54.41%
**Extended Simple Model (C = 2.75)**	23.53%	77.94%	72.06%
**MNI Model (C = 2.55)**	35.29%	64.71%	64.71%
**Extended MNI Model (C = 2.70)**	39.71%	75.00%	79.41%
**HO Subcortical Model (C = 2.30)**	35.29%	67.65%	70.59%
**Extended HO Subcortical Model (C = 2.50)**	35.29%	73.53%	79.41%
**HO Cortical Model (C = 1.00)**	25.00%	60.29%	67.65%
**Extended HO Cortical Model (C = 0.85)**	30.88%	60.29%	60.29%
**Problem-specific Model (C = 2.75)**	41.18%	66.18%	67.65%
**Extended Problem-specific Model (C = 2.55)**	55.88%	82.35%	85.29%
**VLSM Model (C = 2.35)**	25.00%	52.94%	60.29%
**Extended VLSM Model (C = 2.65)**	25.00%	50.00%	61.76%

Overall, it can be seen that the simple SVM model using the lesion volume as the only feature performed worst with an exact accuracy of 23.53%, sliding-window accuracy of 57.36%, and binary accuracy of 54.41%. The extended simple model, which additionally integrates the lesion side, patient age, and admission NIHSS, did not lead to a better exact accuracy but to a considerable improvement of the sliding window accuracy and binary accuracy.

Compared to the results of the two simple models, the MNI SVM models, which make use of the predefined structural MNI brain regions for analysis of the lesion location, led to considerably better results. Again, the extended model, which was generated using the lesion volume, patient age, and admission NIHSS as additional features, performed considerably better compared to the MNI model generated without these additional features. The Harvard-Oxford subcortical SVM models led to similar results compared to the corresponding MNI SVM models. Compared to the MNI and Harvard-Oxford subcortical SVM models the Harvard-Oxford cortical SVM models led to considerably worse classification results, which holds true for all quantitative measures used in this work. The VLSM models, which directly make use of the lesion-based t-score sum instead of using the overlap measurements, did not improve the classification results of the simple models. The extended VLSM model even led to a worse sliding-window and binary accuracy compared to the extended simple model.

The overall best results were obtained for the SVM models using the problem-specific brain regions for overlap quantification in combination with the optional parameters yielding an exact accuracy of 55.88%, sliding-window accuracy of 82.35%, and binary accuracy of 85.35%.

These quantitative results can be also confirmed by the Bland-Altman plots (see S1 Fig). Here, it can be seen that the extended models led to narrower relative limits of agreement in all cases compared to the corresponding models without lesion volume, patient age, and admission NIHSS as additional features. In line with the accuracy results, the extended problem-specific model led to the overall narrowest limits of agreement.

### 3.3. Correlation results

The results of the statistical evaluation of the lesion overlap measurements using Spearman’s rank correlation coefficient are given in Table 4 for the structural MNI brain regions and problem-specific brain regions. The corresponding results of the Harvard-Oxford cortical and subcortical brain regions can be found in S1 Table, but are not discussed here in detail due to the high number of brain regions.

**Table 4:** Correlation coefficients between the follow-up mRS outcome and the three optional parameters (lesion volume, age, and admission NIHSS), the lesion-based t-score sum, as well as lesion overlap measures of the predefined MNI brain structures and automatically determined problem-specific VOIs.

Parameter or Brain Structure	Left	Right
Lesion Volume	0.589 (n = 37, p<0.001)	0.796 (n = 31, p<0.001)
Age	-0.074 (n = 37, p = 0.663)	-0.084 (n = 31, p = 0.654)
Admission NIHSS	0.651 (n = 37, p<0.001)	0.384 (n = 31, p = 0.033)
t-score Sum	0.783 (n = 37, p<0.001)	0.808 (n = 31, p<0.001)
Caudate	0.308 (n = 17, p = 0.230)	0.216 (n = 22, p = 0.334)
Insula	0.656 (n = 30, p<0.001)	0.620 (n = 25, p = 0.001)
Putamen	0.461 (n = 25, p = 0.020)	0.354 (n = 24, p = 0.089)
Thalamus	0.329 (n = 12, p = 0.296)	0.186 (n = 16, p = 0.489)
Cerebellum	- (n = 0)	- (n = 1)
Frontal Lobe	0.142 (n = 20, p = 0.549)	0.644 (n = 21, p = 0.002)
Occipital Lobe	0.044 (n = 10, p = 0.904)	0.512 (n = 7, p = 0.240)
Parietal Lobe	0.224 (n = 22, p = 0.316)	0.741 (n = 18, p<0.001)
Temporal Lobe	0.574 (n = 21, p = 0.007)	0.601 (n = 20, p = 0.005)
Problem-specific VOI 1	0.422 (n = 31, p = 0.018)	0.755 (n = 28, p<0.001)
Problem-specific VOI 2	0.601 (n = 30, p<0.001)	0.628 (n = 29, p<0.001)
Problem-specific VOI 3	0.567 (n = 31, p = 0.001)	0.819 (n = 29, p<0.001)
Problem-specific VOI 4	0.672 (n = 33, p<0.001)	0.642 (n = 27, p< 0.001)

Overall, moderate to strong positive correlations with the mRS score were found for the lesion volume and admission NIHSS parameters. More precisely, a higher correlation between the lesion volume and mRS score was found for right-hemispheric strokes compared to the left-hemispheric strokes (r = 0.796 vs. r = 0.589) while a higher correlation was found for the admission NIHSS parameter in left-hemispheric strokes compared to right-hemispheric strokes (r = 0.651 vs. r = 0.384). No correlation between the patient age and mRS score was found. The lesion-based t-score sum was found to be highly correlated with the mRS score (r = 0.783 / 0.808). However, the t-score sum was also highly correlated with the lesion volume (r = 0.885).

The highest correlations for the predefined MNI atlas brain regions were found for the left and right insula (left hemisphere: r = 0.656 and right hemisphere: r = 0.620). Moderate positive correlations were also found for the left and right putamen (left hemisphere: r = 0.461 and right hemisphere: r = 0.354), for the left and right temporal lobe (left hemisphere: r = 0.574 and right hemisphere: r = 0.601) as well as for the right-hemispheric frontal lobe (r = 0.644), occipital lobe (r = 0.512), and parietal lobe (r = 0.741).

The problem-specific VOIs led to significant higher correlation values compared to the MNI VOIs (two-sided t-test: p = 0.012), Harvard-Oxford subcortical VOIs (p = 0.016) and Harvard-Oxford cortical VOIs (p = 0.008) with moderate to high correlation coefficients ranging between 0.422 and 0.819. Very similar correlation coefficients between 0.601 and 0.672 were found for left and right-hemispheric strokes for the lesion overlap measurements of the second and fourth problem-specific VOIs. In contrast to this finding, higher correlation coefficients were found for right hemispheric strokes between the mRS score and the lesion overlap values of the first and third problem-specifically determined VOI (r = 0.755 / r = 0.819) compared to left-hemispheric strokes (r = 0.422 / r = 0.672).

## Discussion

The method for functional outcome prediction described in the present study is novel due to two main aspects. First, a graded instead of a binary outcome was predicted using a high-level machine learning technique and information about the lesion location in addition to lesion volume and other typically used clinical parameters. Second, problem-specific VOIs, which were determined using the basic principles of voxel-based lesion symptom mapping, were used for lesion overlap quantification resulting in considerable better prediction results compared to the usage of standard predefined brain regions.

Numerous publications focusing on predicting the functional outcome after an ischemic stroke based on clinical and/or image-based features have been presented in the past. Overviews and reviews of these studies can, for example, be found in [21–23] and only a few representative findings are described in the following sections.

Weimar et al. [24], for example, presented a logistic regression model for predicting the long-term functional outcome of acute stroke patients using only clinical features. The features used in this model comprised age, right and left arm paresis, sub-acute NIHSS, sub-acute mRS, gender, previous stroke, lenticulostriate infarction, and neurological complications. Based on an evaluation using a database of 1754 prospectively collected patient records, it was shown that this model achieves an accuracy of 80.7% predicting a good vs. bad outcome, whereas a good outcome was defined by a Barthel Index [25] ≥95 after 100 days. Another logistic regression prediction model using only clinical parameters was presented by Kent et al. [26]. The features used for this model comprise age, acute NIHSS, sex, diabetes, previous stroke, systolic blood pressure, and time from symptom onset to thrombolysis. An evaluation based on 2184 datasets collected from 5 clinical trials revealed that this model is capable of predicting a good outcome (mRS < 2) with an area under the receiver-operator characteristic curve (ROC-AUC) value of 0.788.

Overall, the performance of models using only clinical features to predict a dichotomized functional outcome appears to be limited to an accuracy of maximally 80%. Thus, it is an intuitive idea that the additional inclusion of image-based variables might lead to further improvements of the prediction accuracy. Within this context, it has, for example, been shown that patients with smaller lesions in DWI datasets acquired in the acute phase of a stroke are more likely to reach a favorable outcome [27]. Thus, several studies have been presented in the past using image-based parameters as additional features for functional outcome prediction. For instance, Thijs et al. [28] presented a multi-variable logistic regression prediction model based on the clinical parameters age, initial NIHSS, presence of small-vessel stroke, previous strokes, diabetes, and disability prior to stroke as well the lesion volume determined from DWI datasets acquired at admission as an image-based feature. Apart from this model, two other models using only clinical parameters or only the lesion volume as features were generated and evaluated for comparison purposes. A good outcome was defined by NIHSS < 2, Barthel Index ≥ 95 and Glasgow Outcome Scale = 1, while a very poor outcome was defined by NIHSS ≥ 20, Barthel Index < 60 and Glasgow Outcome Scale > 1. All three feature models were generated and evaluated for each dichotomized outcome variable. Except for the very poor NIHSS dichotomization, the highest ROC-AUC values were found in all cases for the combined models using clinical as well as image-based features (ROC-AUC: 0.79–0.88). A good review of the predictive value of the lesion volume as an additional parameter for prediction of the functional outcome is, for example, given by Schiemanck et al. [29]. Other imaging features, apart from the lesion volume, have also been used as additional predictor variables. Reid et al. [30], for example, found that the best prediction of a good outcome (mRS < 3) can be achieved if using a logistic regression model integrating clinical information about age, pre-stroke independence, arm power, stroke severity score at admission, as well as the two image-based features leukoaraiosis score and presence of focal CT abnormalities determined from CT datasets. Based on a database of 538 patients, an ROC-AUC value of 0.901 was calculated for this model, while the two other models using only clinical parameters tested in this study performed slightly worse (ROC-AUC: 0.882/0.876).

In summary, all previously presented methods for predicting the functional outcome after an ischemic stroke used dichotomized outcome variables instead of graded outcome data. However, the functional outcome within one of the dichotomized groups may differ quite dramatically for the patient, e.g. between mRS 3 and mRS 4. Furthermore, most previous studies used logistic regression modelling instead of high-level machine learning techniques, which may be more powerful. Finally, even though the lesion volume has been used frequently as an additional image-based feature, the lesion location has been used only rarely for functional outcome prediction despite the fact that it has been shown that the combination of the stroke volume and location leads to a better correlation with the functional deficit than the lesion volume alone [9].

Several important conclusions can be made based on the results of the present study. First, the results of the study show that it is beneficial for the prediction performance of all SVM models tested to include information about the lesion location in terms of lesion overlap values in the prediction model. In doing so, the prediction performance improved considerably compared to the two simple SVM models that use only the lesion volume with or without the optional input features. Thus, it may be concluded that the lesion location, as hypothesized, is indeed an important determinant of the functional outcome.

Second, the inclusion of the optional parameters led to a quantitative improvement of the prediction accuracy for all SVM models. This may be an indicator that the functional outcome of an acute ischemic stroke depends on more factors than only lesion volume and location.

Third, the problem-specific VOIs for lesion overlap quantification led to considerably better prediction results compared to the SVM models, which used the predefined structural MNI and Harvard Oxford cortical and subcortical brain regions for overlap quantification. This finding is also supported by the fact that high correlations were found for all problem-specifically determined VOIs but not for all predefined atlas brain regions. Thus, some of these predefined brain regions may be irrelevant or even redundant for the classification precision. Within this context, it has to be pointed out that no initial feature selection, e.g. by F-score-based feature ranking [31], was performed in this study, which might be beneficial to improve the prediction precision. This issue appears to be especially relevant for the Harvard-Oxford cortical atlas, which consists of 48 different brain regions. Due to this fine parcellation, 13 brain regions in the left hemisphere and 7 brain regions in the right hemisphere are only lesioned in three or fewer patients in this cohort, rendering the utility as lesion overlap features for the classification questionable. This assumption is further supported by the poor performance of the corresponding two SVM models compared to the SVM models using the other three brain region definitions. The fact that the SVM models using MNI and Harvard-Oxford subcortical brain regions perform rather similar may be attributed to the fact that both atlases include several brain structures that are associated to motoric functions, which denote a major part of the mRS. The different correlation results found for identical brain structures of the MNI and Harvard-Oxford subcortical atlases can be attributed to different definitions of these structures in the atlas space.

Finally, the results of this study also suggest that a graded prediction of the functional outcome is feasible and does especially benefit from the usage of the problem-specific VOIs for lesion overlap quantification, which were generated using a procedure derived from voxel-based lesion symptom mapping. In contrast to this finding, the VLSM prediction models, which directly use the t-score map, led to considerably worse classification results compared to the problem-specific models and to similar results as the simple models. This finding may be explained by the high correlation between the t-score sum and lesion volume limiting the additional informative value of the t-score feature. A second reason for this finding may be that t-scores were only calculated for voxels that display a lesion in at least five patients. Therefore, lesion portions outside this mask do not contribute towards the t-score sum, which may impair a potentially useful relation of this parameter to the mRS score. In contrast to this approach, those regions are still covered by the problem-specific brain regions by the fourth VOI and, thus, contribute potentially valuable information for the mRS prediction.

It has to be emphasized that the present work has several limitations. First, the study cohort used in the work does not represent a typical distribution of mRS scores. However, the patients used in this study were especially selected to achieve a balanced distribution of mRS scores to prevent a subsequent bias in the SVM model generation. It is, therefore, necessary to further validate the lesion-based mRS prediction method using an independent and representative group of stroke patients. Such a validation using an independent database is also necessary to rule out the possibility of an overfit, which, for example, may result from the optimization of the cost parameter. One drawback of the cost parameter is that it is not intuitive to define. Cost parameters that are too low may lead to underfitting while cost parameters that are too high may lead to overfitting. It has been suggested that the cost parameter should be selected equal or in the range of the number of output classes (6 in this work) to achieve robust results [32]. Since the cost parameter was rather similar for all models (2.3–2.9) except for the two Harvard-Oxford cortical models and smaller than the number of output classes, the risk of a potential overfitting appears rather low. The reason for the considerably lower optimal cost parameters for the two classification models using the Harvard-Oxford cortical brain regions may be caused by the high number of uninformative input features, also leading to the comparably lower classification accuracies.

Within this context, it also important to note that the problem-specific brain regions were generated using the same database as used for training and testing of the classifiers, which may bias the results. However, the leave-one-out evaluation also included the generation of these VOIs by excluding the patient to be classified from the calculation of the voxel-wise statistics to reduce the effect of this bias.

It has to be highlighted that, although the problem-specifically defined brain regions led to better results compared to the usage of the standard MNI or Harvard-Oxford brain regions, the definition itself might still not represent the best-possible choice yet. For example, it is still a matter of debate, which statistical test is best suited for this purpose [33]. Within this context, a Wilcoxon-Mann-Whitney-Test was evaluated in a secondary analysis as an alternative to the t-test, which was used in this study to compute the significance level for each voxel. The statistical maps resulting from these two tests as well as the subsequently determined problem-specific brain regions were very similar. Nevertheless, it has not been evaluated yet if other statistical tests, such as the Brunner-Munzel-Test [33], which have also been suggested and used for this purpose in the past, lead to considerably different results. It should also be mentioned that no correction for multiple comparisons was performed since the p-value map was only used to obtain an initial definition of voxels that may be important for the mRS score. Although very high significance levels were found in some voxels, a correction for multiple comparisons, such as a Bonferroni correction, would have led to a significant smaller initial brain region, which would not cover the majority of the stroke lesions. Thus, such a brain region would not represent a good initial choice for the lesion overlap quantification. Similar to the statistical test itself, the partitioning of the initial segmentation into sub-regions using differences of the median mRS scores might not represent the optimal choice yet, and a more detailed analysis needs to be conducted to identify the best method for the definition of the final brain regions used for overlap quantification and subsequent graded mRS score prediction. Therefore, the problem-specific VOIs determined in this work should not be mistaken as a new gold standard for lesion analysis yet, as more in-depth analyses and validations, for example, within prospective studies, are required. Especially for defining the problem-specific brain regions, a larger database would have been more favorable to reduce the number of voxels that had to be excluded from VLSM calculation due to the insufficient number of lesioned voxels. Likewise, the training of the SVM models would also benefit from a larger database.

Moreover, only patients with a present unilateral infarction in the territory of the middle cerebral artery (MCA) without previous strokes were used for mRS score prediction. Thus, no conclusions regarding the prediction power in case of bilateral strokes or secondary strokes can be made based on this study. Also, the problem-specific brain regions used may not be optimal to predict the functional outcome in case of PCA or ACA strokes. However, MCA strokes are about 5 times more common than isolated ACA and PCA strokes [34] and, thus, clinically more relevant.

Overall, using the multi-class SVM, the best lesion-based mRS score prediction performance was achieved using a combination of the problem-specifically defined brain regions for overlap quantification and patient age, stroke laterality, admission NIHSS, and stroke volume as additional features. With this setup, an exact mRS score prediction accuracy of 56%, a sliding window accuracy of 82%, and binary accuracy of 85% was achieved. Although these numbers seem like a promising result, they also show that the predicted mRS score was more than 1 point different from the ground-truth mRS in 18% of all cases. These cases were rather uniformly distributed among the mRS scores. Thus, another objective of future research is the improvement of the mRS prediction accuracy by optimizing the problem-specifically defined brain regions as well as by including further clinical parameters. The long-term goal is to combine the lesion-based functional outcome prediction described in this work with a prediction of the tissue fate at a voxel-level, for instance, by using multi-parametric statistics or machine-learning techniques.

## Conclusions

In conclusion, a prediction of the graded functional outcome associated with lesion volume and location using high-level machine learning techniques appears feasible but needs to be further validated using a large and representative independent database. The described method may prove especially valuable if combined with voxel-wise tissue outcome predictions based on multi-parametric imaging acquired at the acute stage instead of follow-up imaging, which would allow a real prediction of the future functional outcome.

## Supporting Information

S1 Data Confusion matrices for the twelve mRS prediction models. (PDF)

S1 Fig Bland-Altman plots for the different classification models. (PDF)

S1 Table Correlation coefficients between the follow-up mRS outcome and the lesion overlap measures of the predefined Harvard-Oxford brain structure VOIs. (PDF)
