# Supplementary material for: Multiclass Support Vector Machine-Based Lesion Mapping Predicts Functional Outcome in Ischemic Stroke Patients
Source: PLoS One. 2015 Jun 22;10(6):e0129569. doi: 10.1371/journal.pone.0129569 (PMC4476759; doi:10.1371/journal.pone.0129569)
Supplement: S1 Data — (PDF) [file pone.0129569.s001.pdf]

S1 Data: Confusion matrices for the twelve mRS prediction models

Simple Model

		Predicted					
		mRS 0	mRS 1	mRS 2	mRS 3	mRS 4	mRS 5
Ground Truth	mRS 0	3	1	2	4	0	2
	mRS 1	1	2	1	4	1	3
	mRS 2	1	1	2	5	1	2
	mRS 3	2	1	2	3	3	1
	mRS 4	0	2	2	2	3	3
	mRS 5	0	0	0	1	4	3

Extended Simple Model

		Predicted					
		mRS 0	mRS 1	mRS 2	mRS 3	mRS 4	mRS 5
Ground Truth	mRS 0	2	6	2	2	0	0
	mRS 1	2	2	6	1	0	1
	mRS 2	0	2	2	4	3	1
	mRS 3	1	0	4	0	5	2
	mRS 4	0	1	1	2	4	4
	mRS 5	0	0	0	0	2	6

MNI Model

		Predicted					
		mRS 0	mRS 1	mRS 2	mRS 3	mRS 4	mRS 5
Ground Truth	mRS 0	7	3	2	0	0	0
	mRS 1	3	4	2	0	1	2
	mRS 2	0	3	2	2	4	1
	mRS 3	2	1	2	4	1	2
	mRS 4	1	2	2	1	4	2
	mRS 5	1	1	2	0	1	3

Extended MNI Model

		Predicted					
		mRS 0	mRS 1	mRS 2	mRS 3	mRS 4	mRS 5
Ground Truth	mRS 0	7	3	1	1	0	0
	mRS 1	3	6	2	0	1	0
	mRS 2	1	2	5	1	2	1
	mRS 3	1	1	1	6	2	1
	mRS 4	1	1	1	3	1	5
	mRS 5	1	0	1	2	2	2

Harvard-Oxford Subcortical Model

		Predicted					
		mRS 0	mRS 1	mRS 2	mRS 3	mRS 4	mRS 5
Ground Truth	mRS 0	6	4	1	0	0	1
	mRS 1	6	1	0	1	2	2
	mRS 2	2	1	5	2	0	2
	mRS 3	0	3	1	4	2	2
	mRS 4	0	1	1	2	6	2
	mRS 5	0	1	3	0	2	2

Extended Harvard-Oxford Subcortical Model

		Predicted					
		mRS 0	mRS 1	mRS 2	mRS 3	mRS 4	mRS 5
Ground Truth	mRS 0	6	3	1	0	1	1
	mRS 1	5	4	2	0	1	0
	mRS 2	1	0	8	1	2	0
	mRS 3	2	1	1	3	3	2
	mRS 4	1	0	1	7	2	1
	mRS 5	0	0	2	2	3	1

Harvard-Oxford Cortical Model							
		Predicted					
		mRS 0	mRS 1	mRS 2	mRS 3	mRS 4	mRS 5
Ground Truth	mRS 0	1	6	2	1	1	1
	mRS 1	3	7	0	1	0	1
	mRS 2	4	2	1	3	2	0
	mRS 3	0	2	1	4	2	3
	mRS 4	5	1	1	1	2	2
	mRS 5	0	1	1	0	4	2

Extended Harvard-Oxford Cortical Model							
		Predicted					
		mRS 0	mRS 1	mRS 2	mRS 3	mRS 4	mRS 5
Ground Truth	mRS 0	1	6	1	1	2	1
	mRS 1	3	7	0	1	0	1
	mRS 2	2	2	2	3	2	1
	mRS 3	2	1	2	5	0	2
	mRS 4	4	1	3	0	3	1
	mRS 5	0	0	2	0	3	3

Problem-specific Model							
		Predicted					
		mRS 0	mRS 1	mRS 2	mRS 3	mRS 4	mRS 5
Ground Truth	mRS 0	7	1	1	1	2	0
	mRS 1	5	2	1	1	2	1
	mRS 2	3	2	4	2	0	1
	mRS 3	3	2	2	5	0	0
	mRS 4	1	3	0	2	4	2
	mRS 5	0	1	0	1	0	6

Extended Problem-specific Model							
		Predicted					
		mRS 0	mRS 1	mRS 2	mRS 3	mRS 4	mRS 5
Ground Truth	mRS 0	6	3	3	0	0	0
	mRS 1	4	7	0	1	0	0
	mRS 2	2	1	8	1	0	0
	mRS 3	1	0	4	4	2	1
	mRS 4	1	1	0	3	7	0
	mRS 5	0	1	0	1	0	6

VLSM Model							
		Predicted					
		mRS 0	mRS 1	mRS 2	mRS 3	mRS 4	mRS 5
Ground Truth	mRS 0	7	1	1	1	2	0
	mRS 1	5	2	1	1	2	1
	mRS 2	3	2	4	2	0	1
	mRS 3	3	2	2	5	0	0
	mRS 4	1	3	0	2	4	2
	mRS 5	0	1	0	1	0	6

Extended VLSM Model							
		Predicted					
		mRS 0	mRS 1	mRS 2	mRS 3	mRS 4	mRS 5
Ground Truth	mRS 0	6	5	0	0	1	0
	mRS 1	4	5	2	0	0	1
	mRS 2	6	4	0	0	2	0
	mRS 3	6	1	0	3	0	2
	mRS 4	8	1	1	0	1	1
	mRS 5	3	2	0	0	1	2
