# Supplementary material for: Multiclass Support Vector Machine-Based Lesion Mapping Predicts Functional Outcome in Ischemic Stroke Patients
Source: PLoS One. 2015 Jun 22;10(6):e0129569. doi: 10.1371/journal.pone.0129569 (PMC4476759; doi:10.1371/journal.pone.0129569)
Supplement: S1 Fig — (PDF) [file pone.0129569.s002.pdf]

S1 Fig: Bland–Altman plots for the different classification models

(The 95% limits of agreement are shown as two dotted lines)

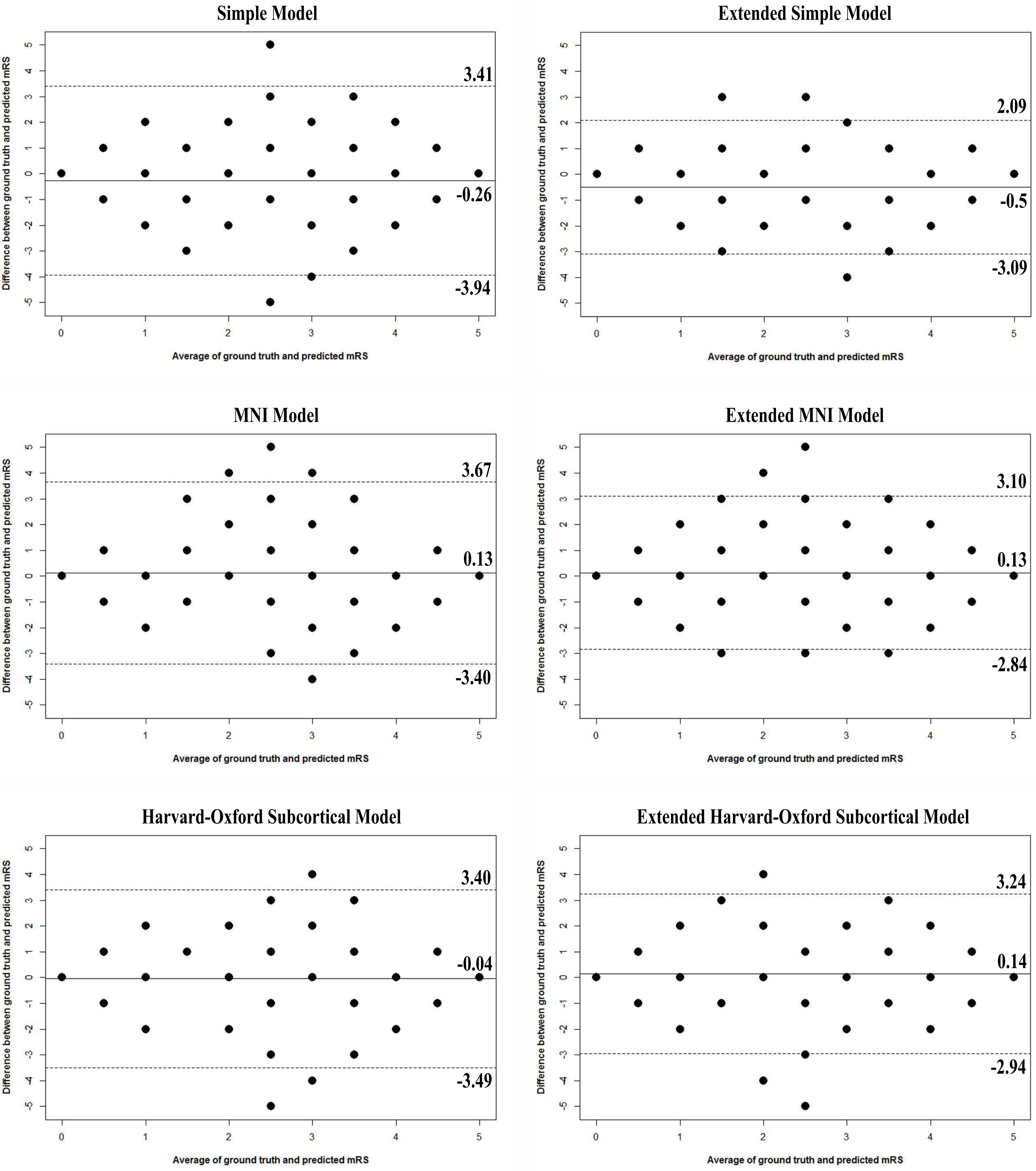

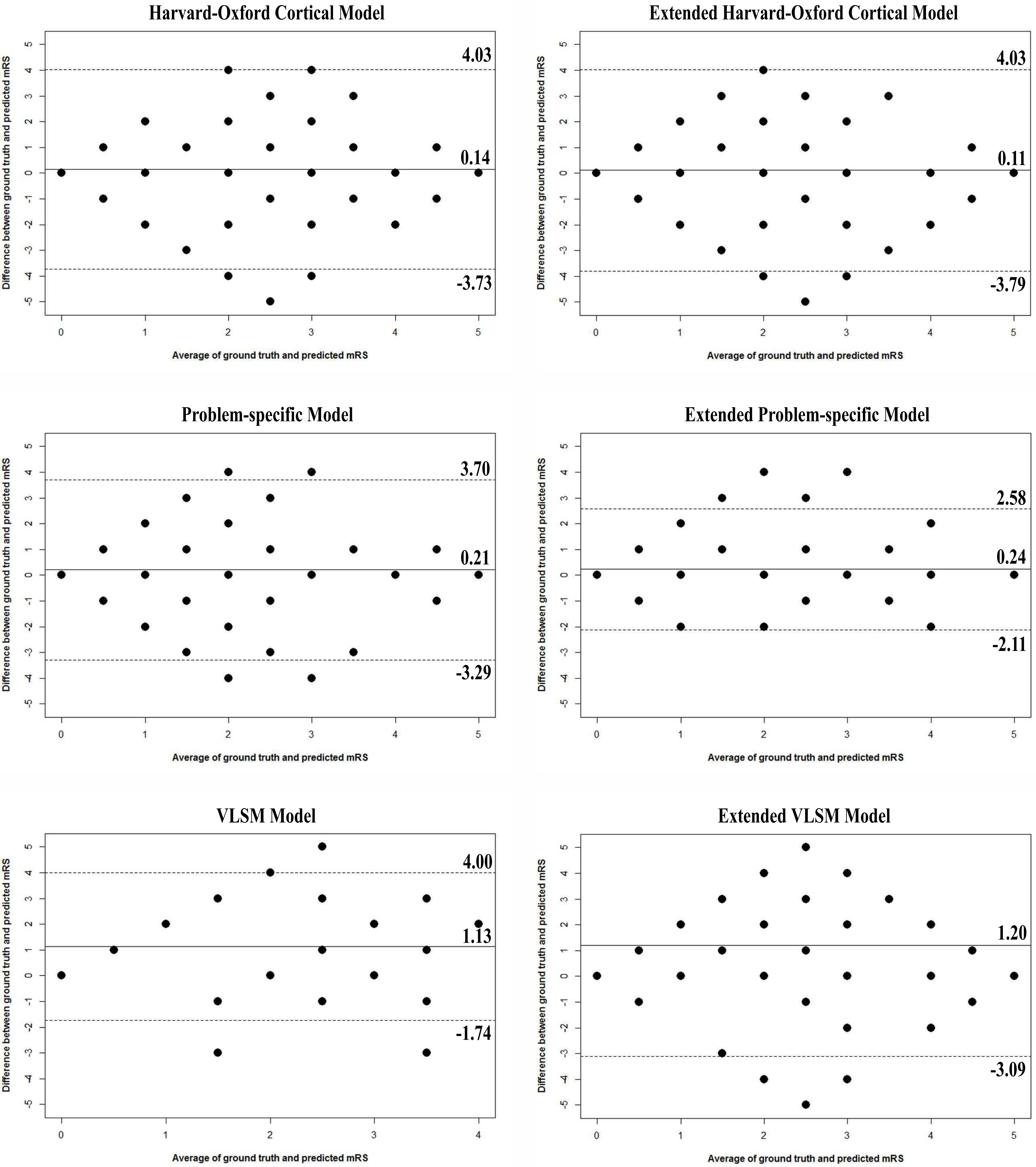

Harvard-Oxford Cortical Model
4.03
0.14
-3.73
Extended Harvard-Oxford Cortical Model
4.03
0.11
-3.79
Problem-specific Model
3.70
0.21
-3.29
Extended Problem-specific Model
2.58
0.24
-2.11
VLSM Model
4.00
1.13
-1.74
Extended VLSM Model
1.20
-3.09
Difference between ground truth and predicted mRS
Average of ground truth and predicted mRS
