# Supplementary material for: Multiclass Support Vector Machine-Based Lesion Mapping Predicts Functional Outcome in Ischemic Stroke Patients
Source: PLoS One. 2015 Jun 22;10(6):e0129569. doi: 10.1371/journal.pone.0129569 (PMC4476759; doi:10.1371/journal.pone.0129569)
Supplement: S1 Table — (PDF) [file pone.0129569.s003.pdf]

Harvard-Oxford Subcortical Brain Structure	Left	Right
Cerebral White Matter	0.623 (n=36, p<0.001)	0.799 (n=29, p<0.001)
Cerebral Cortex	0.296 (n=28, p=0.126)	0.682 (n=27, p<0.001)
Thalamus	0.147 (n=7, p=0.753)	-0.538 (n=7, p=0.213)
Caudate	-0.636 (n=10, p=0.048)	0.093 (n=14, p=0.752)
Putamen	0.285 (n=24, p=0.177)	0.409 (n=23, p=0.053)
Pallidum	0.028 (n=11, p=0.934)	0.257 (n=14, p=0.374)
Hippocampus	0.335 (n=5, p=0.581)	0.347 (n=7, p=0.445)
Amygdala	0.846 (n=10, p=0.002)	0.301 (n=9, p=0.430)
Accumbens	- (n=1)	-0.600 (n=4, p=0.400)
Brain Stem	- (n=0)	- (n=0)
**Harvard-Oxford Cortical Brain Structure**	**Left**	**Right**
Frontal Pole	- (n=2)	-0.698 (n=6, p=0.123)
Insular Cortex	0.632 (n=29, p<0.001)	0.642 (n=25, p=0.001)
Superior Frontal Gyrus	0.738 (n=5, p=0.155)	-0.260 (n=8, p=0.952)
Middle Frontal Gyrus	-0.352 (n=13, p=0.239)	0.460 (n=16, p=0.073)
Inferior Frontal Gyrus - pars triangularis	-0.009 (n=11, p=0.978)	0.542 (n=14, p=0.045)
Inferior Frontal Gyrus - pars opercularis	-0.181 (n=16, p=0.503)	0.416 (n=18, p=0.086)
Precentral Gyrus	0.175 (n=19, p=0.475)	0.692 (n=20, p=0.001)
Temporal Pole	0.590 (n=11, p=0.056)	0.621 (n=13, p=0.024)
Superior Temporal Gyrus - anterior division	0.511 (n=8, p=0.196)	0.798 (n=10, p=0.006)
Superior Temporal Gyrus - posterior division	0.430 (n=14, p=0.125)	0.746 (n=11, p=0.008)
Middle Temporal Gyrus - anterior division	0.335 (n=7, p=0.463)	0.354 (n=5, p=0.559)
Middle Temporal Gyrus - posterior division	0.838 (n=9, p=0.005)	0.000 (n=5, p=1.000)
Middle Temporal Gyrus - temporooccipital part	0.274 (n=9, p=0.476)	0.393 (n=6, p=0.441)
Inferior Temporal Gyrus - anterior division	-0.304 (n=6, p=0.558)	- (n=2)
Inferior Temporal Gyrus - posterior division	0.034 (n=6, p=0.949)	0.354 (n=5, p=0.559)
Inferior Temporal Gyrus - temporooccipital part	- (n=2)	0.676 (n=6, p=0.140)
Postcentral Gyrus	0.389 (n=15, p=0.152)	0.402 (n=16, p=0.122)
Superior Parietal Lobule	0.281 (n=7, p=0.542)	0.270 (n=12, p=0.396)
Supramarginal Gyrus - anterior division	0.102 (n=13, p=0.741)	0.287 (n=12, p=0.367)
Supramarginal Gyrus - posterior division	0.318 (n=14, p=0.268)	0.506 (n=12, p=0.093)
Angular Gyrus	0.251 (n=11, p=0.456)	0.538 (n=11, p=0.088)
Lateral Occipital Cortex - superior division	0.433 (n=9, p=0.244)	0.663 (n=9, p=0.051)
Lateral Occipital Cortex - inferior division	0.441 (n=6, p=0.381)	0.808 (n=7, p=0.028)
Intracalcarine Cortex	0.224 (n=5, p=0.718)	-0.338 (n=6, p=0.512)
Frontal Medial Cortex	- (n=0)	- (n=0)
Juxtapositional Lobule Cortex	0.866 (n=5, p=0.058)	- (n=3)
Subcallosal Cortex	- (n=0)	-0.316 (n=4, p=0.684)
Paracingulate Gyrus	- (n=0)	- (n=0)
Cingulate Gyrus - anterior division	- (n=3)	- (n=3)
Cingulate Gyrus - posterior division	- (n=3)	-0.632 (n=4, p=0.368)
Precuneous Cortex	0.283 (n=9, p=0.460)	0.507 (n=6, p=0.305)
Cuneal Cortex	- (n=2)	0.354 (n=5, p=0.559)
Frontal Orbital Cortex	0.475 (n=10, p=0.165)	0.044 (n=16, p=0.871)
Parahippocampal Gyrus - anterior division	0.717 (n=11, p=0.013)	0.309 (n=6, p=0.552)
Parahippocampal Gyrus - posterior division	-0.224 (n=5, p=0.718)	0.316 (n=4, p=0.684)
Lingual Gyrus	- (n=2)	0.000 (n=5, p=1.000)
Temporal Fusiform Cortex - anterior division	-0.707 (n=5, p=0.182)	- (n=2)
Temporal Fusiform Cortex - posterior division	-0.169 (n=6, p=0.749)	0.894 (n=5, p=0.041)
Temporal Occipital Fusiform Cortex	- (n=3)	- (n=2)
Occipital Fusiform Gyrus	- (n=2)	-0.205 (n=5, p=0.741)
Frontal Operculum Cortex	0.180 (n=17, p=0.490)	0.349 (n=19, p=0.143)
Central Opercular Cortex	0.354 (n=26, p=0.076)	0.417 (n=22, p=0.054)
Parietal Operculum Cortex	0.355 (n=18, p=0.148)	0.248 (n=14, p=0.394)
Planum Polare	0.599 (n=14, p=0.024)	0.637 (n=16, p=0.008)
Heschls Gyrus	0.030 (n=17, p=0.908)	0.280 (n=16, p=0.293)
Planum Temporale	0.164 (n=17, p=0.528)	0.513 (n=15, p=0.050)
Supracalcarine Cortex	- (n=3)	0.000 (n=5, p=1.000)
Occipital Pole	- (n=2)	-0.200 (n=4, p=0.800)

S1 Table: Correlation coefficients between the follow-up mRS outcome and the lesion overlap measures of the predefined Harvard-Oxford brain structure VOIs.
